# Supplementary material for: Enriched environment enhances β‐adrenergic signaling to prevent microglia inflammation by amyloid‐β
Source: EMBO Mol Med. 2018 Aug 9;10(9):e8931. doi: 10.15252/emmm.201808931 (PMC6127891; doi:10.15252/emmm.201808931)
Supplement: Supplementary file 1 — Appendix [file EMMM-10-e8931-s001.pdf]

Enriched environment enhances b-adrenergic signaling to prevent microglia inflammation by amyloid-b

Huixin Xu, Molly M. Rajsombath, Pia Weikop, Dennis J. Selkoe

Appendix

Table of contents:

1. Figure S1. Cytokine profile of human AD brain extract vs immunodepleted and control brain extract

2. Figure S2. FITC-Aβ42 distributes equally in both ventricles after unilateral i.c.v. injection.

3. Figure S3-S5. Microglia images presented with split channels corresponding to Figure 1, 3, and 6.

4. Figure S6. The full blot view of b2AR western blot presented in Figure 7.

5. Table S1-S10. Exact *p* values from each main figure and Expanded View figure.

Figure S1.

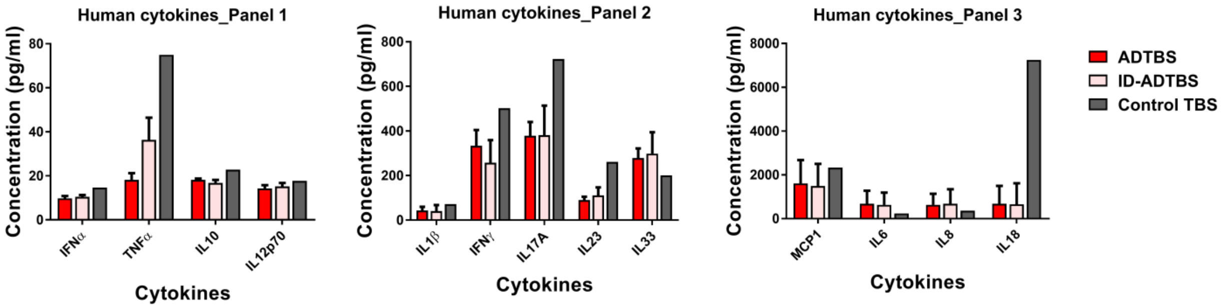

Figure S1. LEGNEDplex^{TM} analysis showed no significant difference between ADTBS and ID-ADTBS. Control TBS extracts from healthy human brain contained no less cytokines than TBS extracts from AD patients' brains.

Figure S2.

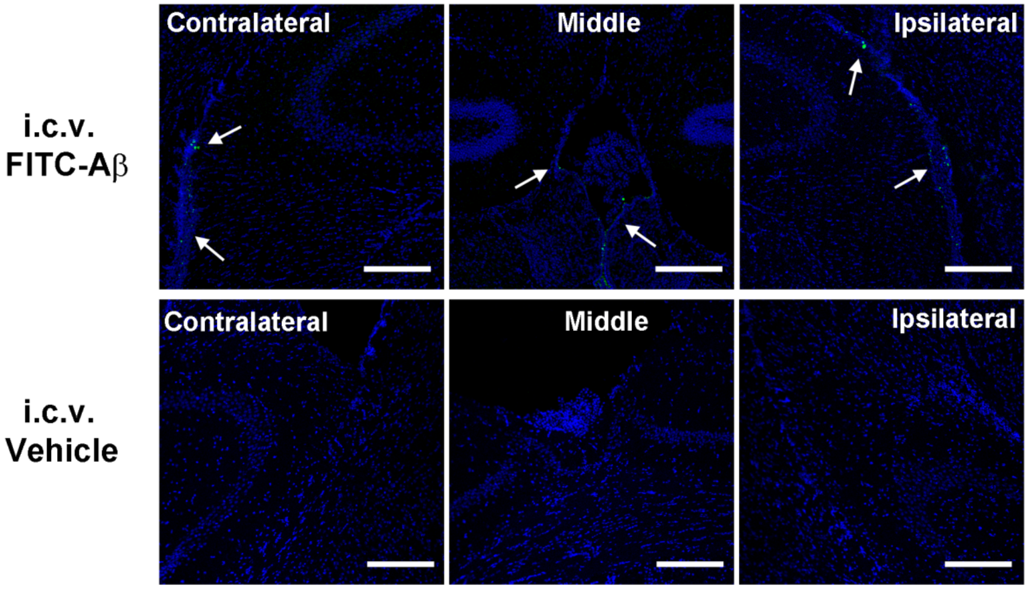

Figure S2. Confocal images on brain sections post intracerebral ventricular injection of FITC-Aβ. FITC signal (green) can be detected on ipsilateral and contralateral ventricles as well as the middle region, as pointed out by white arrows. Brains receiving only vehicle injection showed no positive FITC signals. Scale bar = 200 μm.

Figure S3.

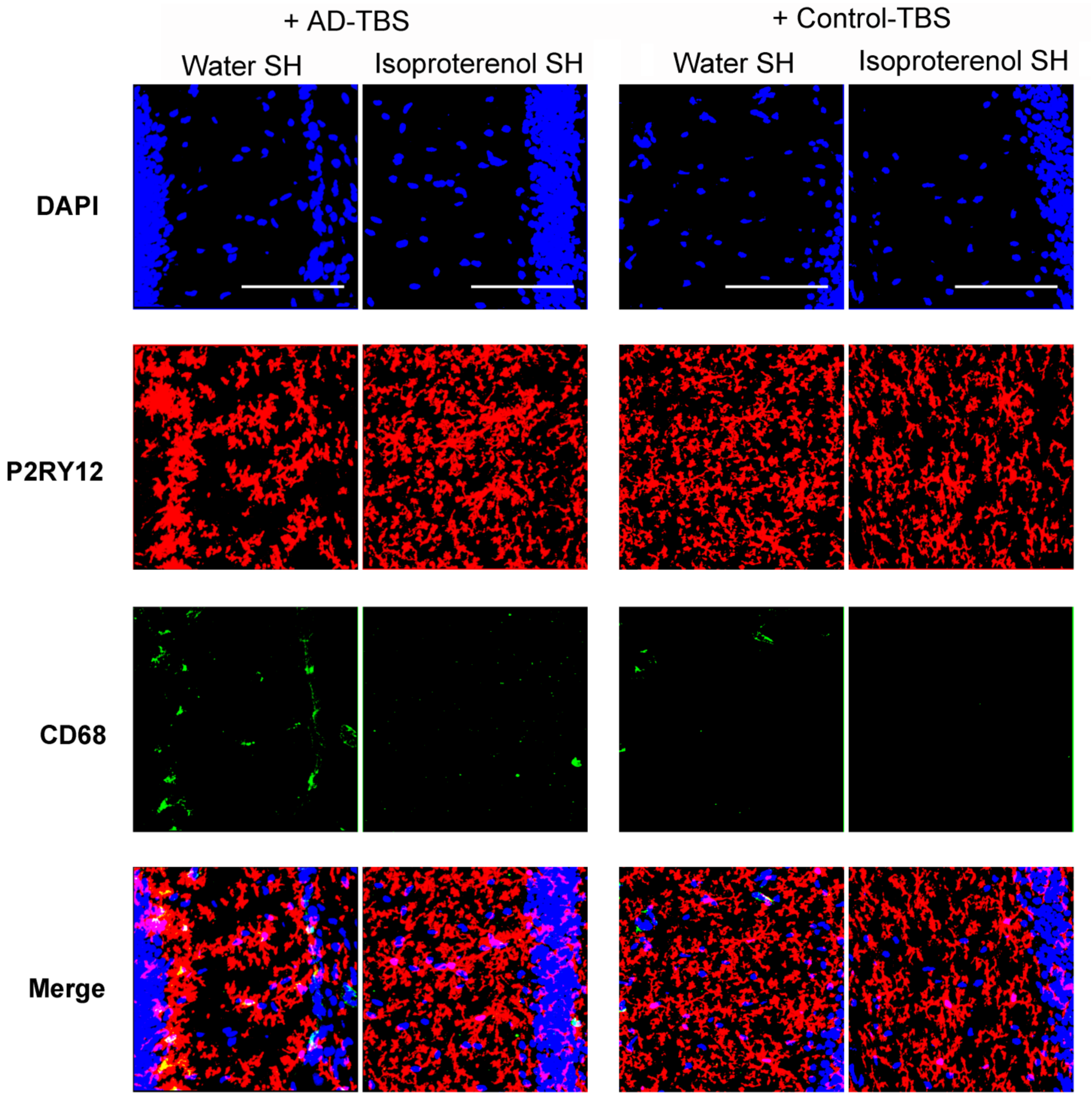

Figure S3. Display of single channel and merged images from panels presented in Figure 1. Scale bar = 100 μm (same in all panels).

Figure S4.

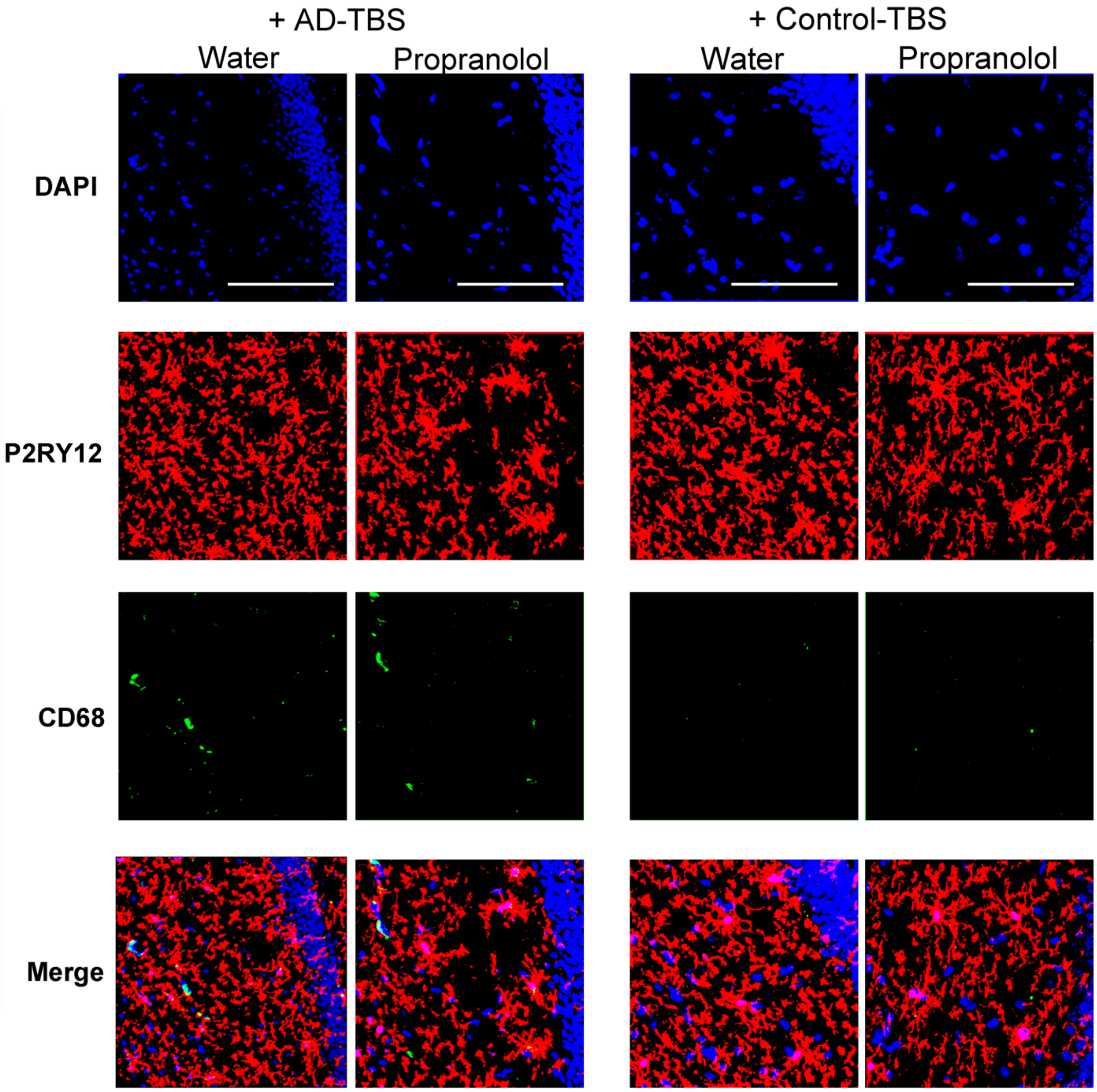

Figure S4. Display of single channel and merged images from panels presented in Figure 3. Scale bar = 100 μm (same in all panels).

Figure S5.

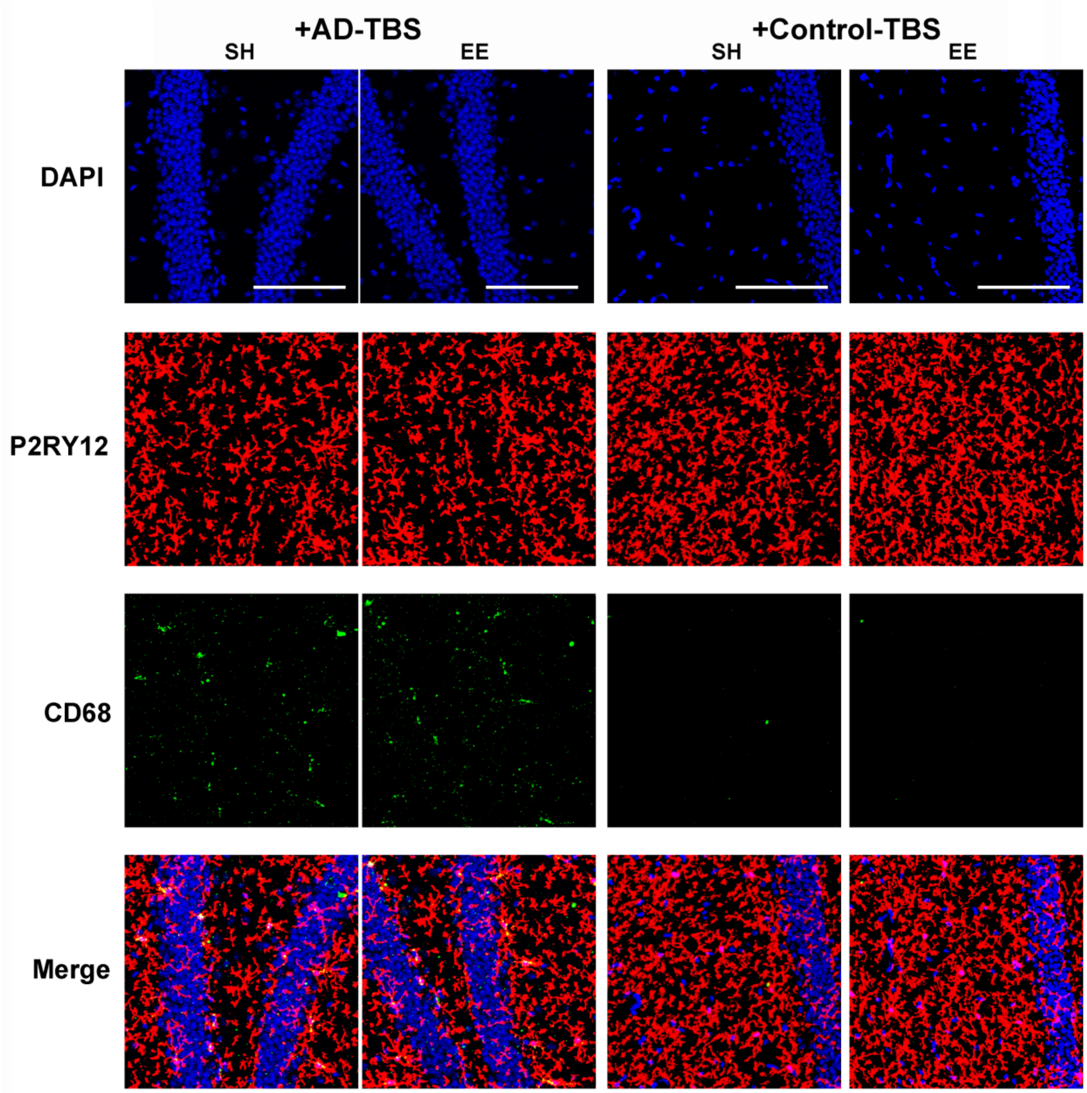

Figure S5. Display of single channel and merged images from panels presented in Figure 6. Scale bar = 100 μm (same in all panels).

Figure S6.

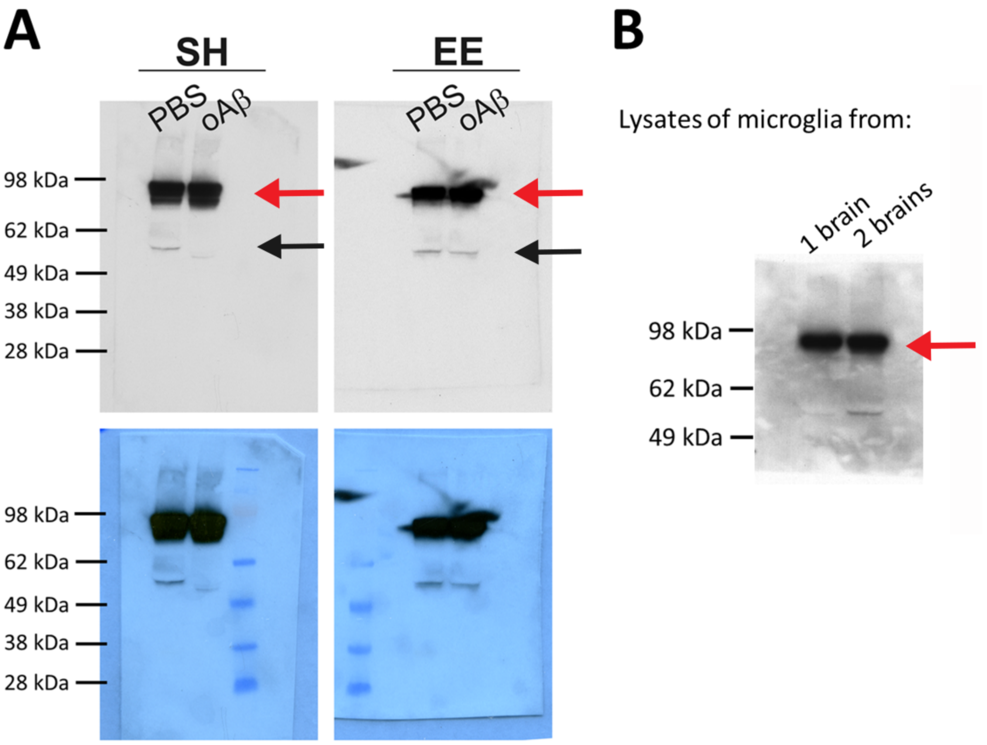

Figure S6. A) Original western blots of β2AR shows bands (black arrow) at correct molecular weight zone. The protein is predicted as 46 kDa but migrates between 50 kDa and 75 kDa as Abcam datasheet shows. Bottom panels were acquired by overlaying developed films with blots to visualize molecular weight markers. B) The high intensity bands at higher molecular weight (red arrow) are non-specific signals that do not respond to changes in loading amount (total microglia cell lysates prepared from 1 brain vs 2 brains).

Table S1. *p* values from Figure 1C. Results not significant (*p*>0.05) are not listed.

	SH water (AD/ctrl)	SH isoproterenol (AD/Ctrl)	Water/Isoproterenol (Ctrl)	Water/Isoproterenol (AD)
#microglia/mm^2	2.93E-06	/	3.48E-06	3.96E-08
Circularity	0.028	/	/	0.0057
Solidity	0.012	/	/	0.0043
%CD68/microglia	0.0026	/	/	0.011
#Branch/microglia	0.0024	/	/	0.0017

Table S2. *p* values from Figure 2B&C.

**Gene**	**Correction**	***p***	**Gene**	**Correction**	***p***
Mef2a	Yes	1.29E-06	Myd88	No	0.0011
Ccl2	Yes	2.51E-07	Ripk2	No	0.0039
Tnf	Yes	2.46E-05	Tlr3	No	0.0019
Ifi44	Yes	3.33E-05	Cxcl10	No	0.0043
Ccl4	Yes	2.06E-07	Nfatc3	No	0.0024
Ccl3	Yes	4.94E-06	Mx1	No	0.0030
Rock2	Yes	0.00017	Nr3c1	No	0.0022
Tlr4	Yes	1.58E-05	Mapk8	No	0.0041
Prkcb	Yes	0.00045	Map3k5	No	0.0030
Il18	Yes	2.8E-06	Cysltr1	No	0.0023
Ptger3	Yes	1.12E-05	Myc	No	0.0194
C1qb	Yes	5.01E-06	Tlr1	No	0.022
Raf1	Yes	0.000207	Tcf4	No	0.0048
Mapkapk2	Yes	4.68E-05	Il10rb	No	0.00158
Jun	Yes	2.77E-05	Il1a	No	0.016
Tlr6	Yes	3.48E-06	Stat1	No	0.00094
Irf1	Yes	4.85E-06	Tlr9	No	0.00069
Gnaq	Yes	0.00018	Irf5	No	0.013
Mef2c_Mm	Yes	0.00010	Stat2	No	0.0037
Map2k1	Yes	8.98E-05			
Cdc42	Yes	1.64E-05			
Ptgs1	Yes	9.18E-06			
Nfe2l2	Yes	6.44E-05			
Tgfbr1	Yes	1.35E-05			
Rhoa	Yes	0.00014			
Myl2	Yes	0.00022			
C1qa	Yes	5.26E-05			
Tgfb1	Yes	0.00026			
Stat3	Yes	0.00018			

Table S3. *p* values from Figure 3C. Results not significant (p>0.05) are not listed.

	EE water (AD/ctrl)	EE propranolol (AD/Ctrl)	Water/Propranolol (Ctrl)	Water/Propranolol (AD)
#microglia/mm^2	/	5.09E-06	3.86E-05	8.12E-08
Circularity	0.00058	0.00032	/	0.0018
Solidity	/	0.00060	/	0.0043
%CD68/microglia	0.0011	0.00071	/	0.011
#Branch/microglia	0.0024	/	/	0.0017

Table S4. *p* values from Figure 4B&C.

**Gene**	**Correction**	***p***	**Gene**	**Correction**	***p***
H2-Eb1	Yes	6.79E-07	Iigp1	No	0.00070
Ifi27l2a	Yes	3.85E-06	Irf7	No	0.00077
Stat1	Yes	1.09E-05	Ifit2	No	0.00071
Ifit3	Yes	6.75E-05	Ccl24	No	0.0034
Ccl4	Yes	1.58E-05	Ifi44	No	0.00056
Ccl3	Yes	0.000321	Cxcl10	No	0.0012
Ly96	Yes	2.97E-05	Ddit3	No	0.0057
Tlr3	Yes	0.00011	Irf1	No	0.020
Ccl2	Yes	7.62E-05	Ltb	No	0.00099
Tyrobp	Yes	2.95E-05	Stat2	No	0.011
Stat3	Yes	7.08E-06	Tnf	No	0.012
Mafg	Yes	1.14E-05	Ptger3	No	0.010
Mef2a	Yes	0.00013	C4a	No	0.0012
			Trem2	No	0.0015
			Gnas	No	0.0025
			Limk1	No	0.00049
			Myc	No	0.0017
			Hmgb2	No	0.0014
			Hdac4	No	0.012

Table S5. *p* values from Figure 5A&B.

**SH water vs isoproterenol**		**EE water vs propranolol**	
**Gene**	***p***	**Gene**	***p***
Ccl2	0.00095	Ccl2	1.25E-08
Ccl3	4.39E-05	Ccl3	3.61E-05
Ccl4	7.33E-05	Ccl4	1.73E-07
Cxcl10	1.06E-10	Cxcl10	0.0016
Tnf	3.65E-06	Tnf	1.34E-09

Table S6. *p* values from Figure 6B. Results not significant (p>0.05) are not listed.

	SH AD/Ctrl	EE AD/Ctrl
#microglia/mm^2	0.0089	0.0011
Circularity	0.00014	0.019
Solidity	0.046	0.017
%CD68/microglia	0.0088	0.019
#Branch/microglia	0.0070	0.00047

Table S7. *p* values from Figure 7F. Results not significant (p>0.05) are not listed.

	**DMEM/oAβ**	**oAβ/oAβ+iso**	**oAβ+iso / oAβ+iso+cAMP-Rp**
Ccl2	0.0035	0.0045	/
Ccl3	0.0044	0.00020	/
Ccl4	0.0016	5.67E-05	/
Cxcl10	0.028	0.0068	/
Tnf	0.0066	0.00032	0.0038

Table S8. *p* values from Figure EV2.

**Cytokine**	***p***
CCL2	0.001547
CCL3	0.0071772
CCL4	0.0141576
CXCL10	0.0057565

Table S9. *p* values from Figure EV3.

**Gene**	***p***
Ccl2	3.07E-07
Ccl3	2.10E-05
Ccl4	6.22E-05
Cxcl10	0.028
Tnf	0.0015

Table S10. *p* values from Figure EV5.

	SH AD/Ctrl	EE AD/Ctrl
Circularity	0.0040	0.0016
Solidity	0.0059	0.037
%CD68/microglia	0.0022	0.0072
#Branch/microglia	0.0022	0.0012
