# Supplementary material for: Enriched environment enhances β‐adrenergic signaling to prevent microglia inflammation by amyloid‐β
Source: EMBO Mol Med. 2018 Aug 9;10(9):e8931. doi: 10.15252/emmm.201808931 (PMC6127891; doi:10.15252/emmm.201808931)
Supplement: Supplementary file 2 — Expanded View Figures PDF [file EMMM-10-e8931-s002.pdf]

Expanded View Figures

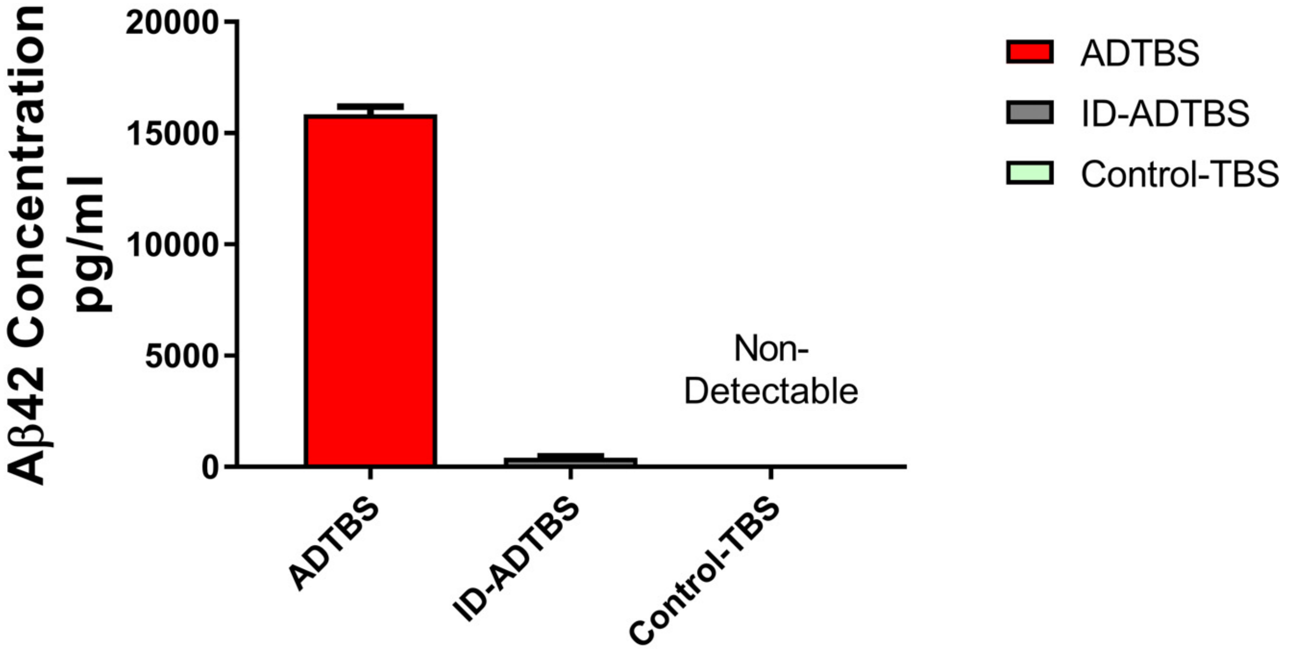

Figure EV1. Control-TBS has no detectable Aβ42, and ID-ADTBS contains less than 5% of the Aβ42 in the original ADTBS.

Values were calculated based on six technical replicates from three independent ELISAs. All data are means ± SD.

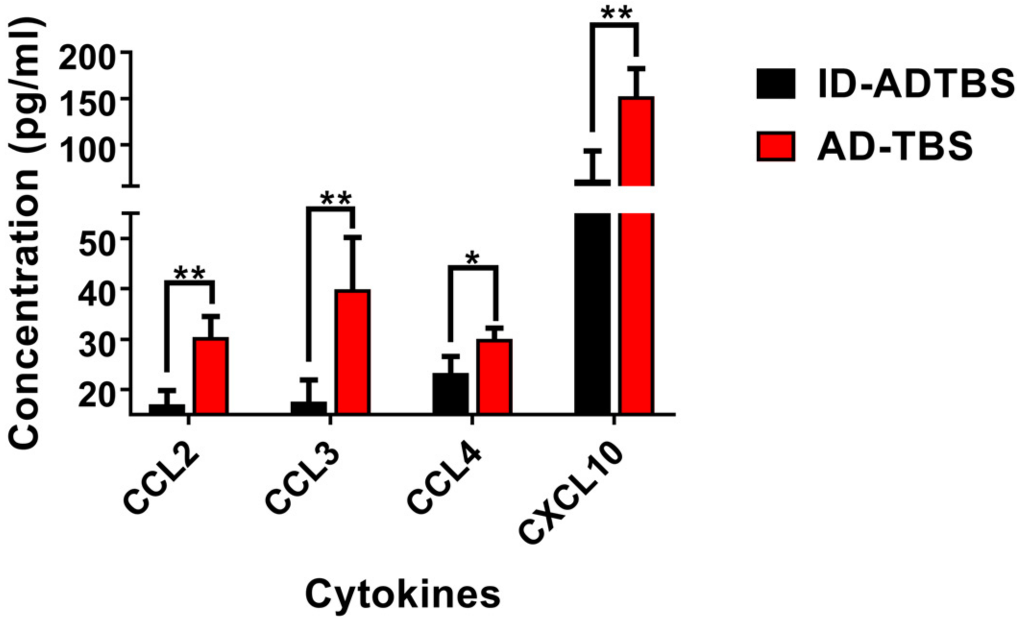

Figure EV2. ADTBS significantly increases the protein levels of cytokines in brain tissue.

$^{*}P < 0.05$, $^{**}P < 0.01$. $N = 5$. All quantitative data are mean ± SD. Exact P values are listed in Appendix Table S8. All statistical analysis were performed using multiple t-test (unpaired, do not assume equal SDs) with Holm-Sidak method to determine significance.

 © 2018 The Authors

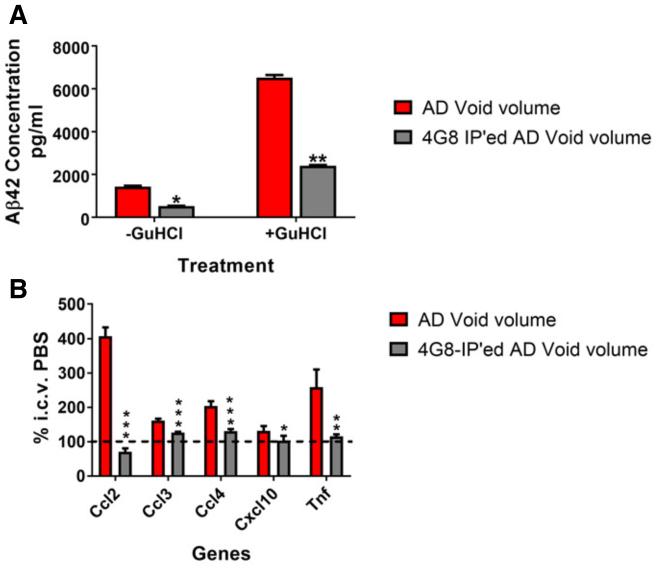

Figure EV3. SEC void volume of AD brain extracts induces Aβ-specific inflammatory responses in mouse microglia.

A Aβx-42 ELISA shows significant removal of Aβ42 from the SEC void volume by immunoprecipitation with 4G8 antibody.

B i.c.v. injection in mice indicates inflammatory responses of five genes induced by SEC-purified AD brain extract with lack of alteration (set at 100%) using Aβ−immunodepleted material.

Data information: $N = 5$. *$P < 0.05$, **$P < 0.01$, ***$P < 0.0001$. All data are means ± SD. Exact P values are listed in Appendix Table S9. All statistical analysis were performed using multiple t-test (unpaired, do not assume equal SDs) with Holm-Sidak method to determine significance.

Figure EV4. SEC-purified oAβ induces consistent microglial gene expression changes in the SH isoproterenol vs. water mouse cohorts and the EE propranolol vs. water mouse cohorts.

$N = 6$. The heap map is plotted with Log_2(oAβ/PBS ratio). The exact ratio values with matching gene names are presented in the Dataset EV3.

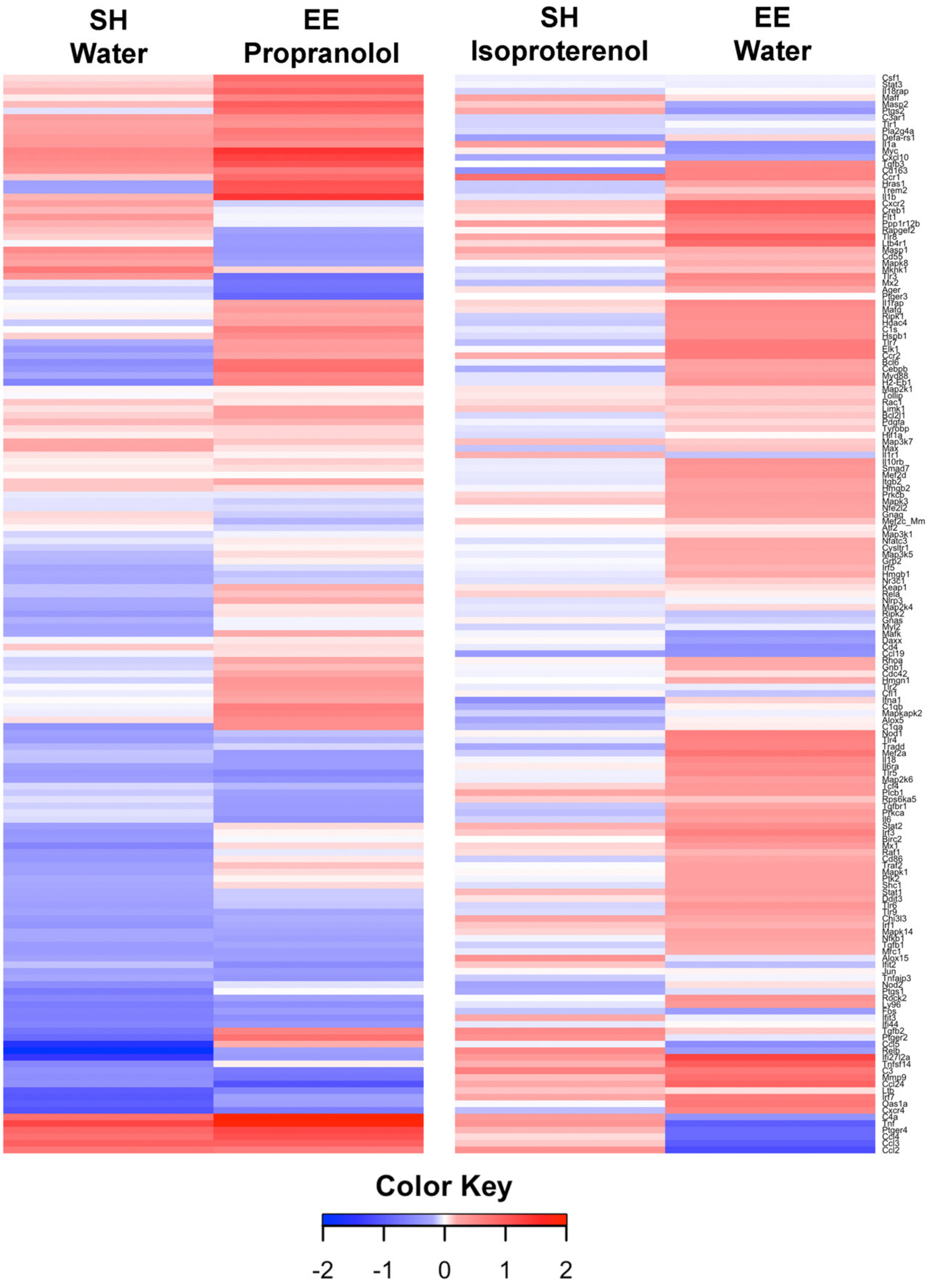

Figure EV4.

© 2018 The Authors

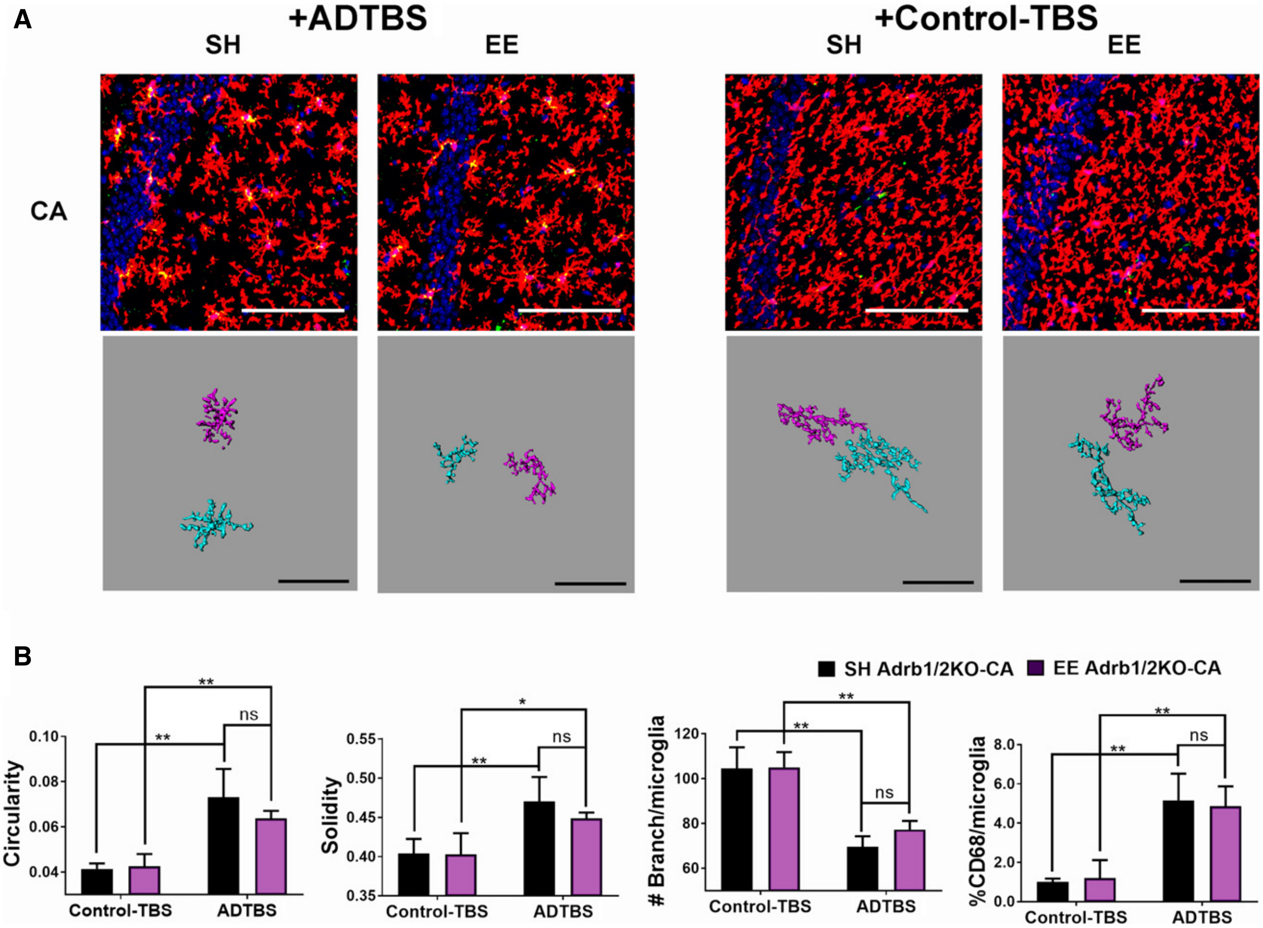

Figure EV5. In Adrb1/2 KO mice, microglia in the CA region have the same degree of morphological changes in SH and EE mice after ADTBS i.c.v. injection.

A Representative images in CA of hippocampus from SH and EE mice injected with ADTBS and control-TBS. When injected with ADTBS, both SH and EE mice have microglia with significant inflammatory morphological features. When injected with control-TBS, both SH and EE mice show minimal microglia inflammatory features. Red: P2ry12; green: CD68; blue: DAPI. Scale bar = 100 μm.

B Quantification of microglia morphology in CA. Both SH and EE Adrb1/2KO mice had significantly increased microglia circularity, solidity, and %CD68, while decreased branching complexity after ADTBS injection compared to their controls (* $P < 0.05$; ** $P < 0.01$; $N = 5$). Exact *P* values are listed in Appendix Table S10. All statistical analysis were performed using multiple *t*-test (unpaired, do not assume equal SDs) with Holm-Sidak method to determine significance.

© 2018 The Authors
